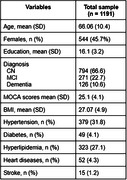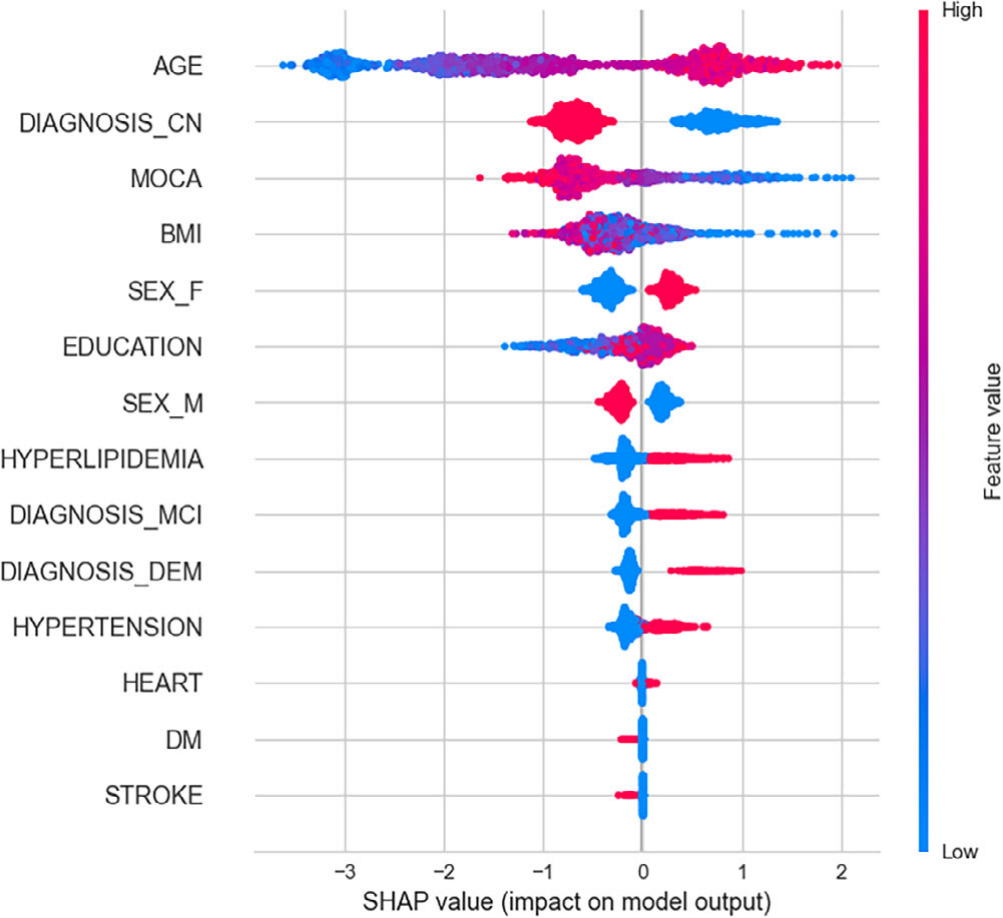# A machine learning approach to predict tau positivity using clinical features in amyloid‐positive individuals

**DOI:** 10.1002/alz.088325

**Published:** 2025-01-09

**Authors:** Daniel Arnold, Wyllians Vendramini Borelli, Luiza Santos Machado, Nesrine Rahmouni, Joseph Therriault, Stijn Servaes, Jenna Stevenson, Arthur C. Macedo, Artur Francisco Schumacher‐Schuh, Christian Mattjie, Rodrigo C. Barros, Marco Antônio de Bastiani, Firoza Z Lussier, Mira Chamoun, Gleb Bezgin, Andrea L. Benedet, Tharick Pascoal, Pedro Rosa‐Neto, Eduardo R. Zimmer, Alzheimer's Disease Neuroimaging Initiative (ADNI)

**Affiliations:** ^1^ Federal University of Rio Grande do Sul, Porto Alegre, Rio Grande do Sul Brazil; ^2^ McGill University, Montreal, QC Canada; ^3^ Translational Neuroimaging Laboratory, The McGill University Research Centre for Studies in Aging, Montréal, QC Canada; ^4^ HCPA, Porto Alegre, Rio Grande do Sul Brazil; ^5^ PUCRS, Porto Alegre, Rio Grande do Sul Brazil; ^6^ University of Pittsburgh, Pittsburgh, PA USA; ^7^ Department of Psychiatry and Neurochemistry, Institute of Neuroscience and Physiology, The Sahlgrenska Academy, University of Gothenburg, Mölndal, Gothenburg Sweden; ^8^ Federal University of Rio Grande do Sul (UFRGS), Porto Alegre, RS Brazil; ^9^ Laboratory of Neuro Imaging (LONI), University of Southern California, Los Angeles, CA USA

## Abstract

**Background:**

Anti‐amyloid therapy appears to have an increased effect on reducing cognitive decline in amyloid‐ and tau‐positive individuals. However, clinical trials inclusion criteria require solely amyloid positivity. Herein, we developed a machine‐learning prediction model to identify tau positivity in amyloid‐positive individuals using clinical variables.

**Method:**

We selected 1191 amyloid‐positive participants and tau status from ADNI, TRIAD, PPMI databases. Commonly shared clinical features selected between datasets were age, total MOCA scores, clinical diagnosis, sex, education, BMI, heart disease, stroke, hyperlipidemia and diabetes. Amyloid positivity was defined by amyloid‐PET (PIB‐PET, FBB‐PET or AZD4694‐PET) or CSF AB42 and Tau positivity defined by Tau‐PET (MK6240‐PET or AV1451‐PET) or CSF p‐tau181. The dataset was split into training (49%), validation (21%), and testing datasets (30%). A XGBoost model was tuned, and then used to predict the tau status outcome in the testing dataset.

**Result:**

A total of 647 men and 544 women were included with mean age 66.1 ± 10.4 (mean ± SD) years, MOCA 25.1 ± 4.1 scores and education 16.1 ± 3.2 years (Table). The population consisted of 794 controls, 271 MCI and 126 dementia individuals. Tau status was negative for 854 individuals and positive for 337. The receiver‐operator characteristic (ROC) analysis showed that the area under the curve (AUC) was 0.81 for discriminating tau‐negative vs. tau‐positive, with 0.76 as sensitivity, specificity as 0.86, and accuracy as 0.83. The top 3 most impactful features were found to be age, diagnosis‐CN and MOCA score through a SHAP value analysis (Figure).

**Conclusion:**

Predicting tau positivity with clinical and cognitive variables may improve the selection of individuals for AD trials. A two‐step workflow using this approach may significantly reduce the need for tau‐PET exams in trials by screening individuals using clinical variables. The following steps should include adding more individuals and other clinical variables through different cohorts to optimize the model applicability and test their performance in outcomes of clinical trials.